# Association Between Daily Worry, Pathological Worry, and Fear of Progression in Patients With Cancer

**DOI:** 10.3389/fpsyg.2021.648623

**Published:** 2021-08-11

**Authors:** Andreas Dinkel, Birgitt Marten-Mittag, Katrin Kremsreiter

**Affiliations:** ^1^Department of Psychosomatic Medicine and Psychotherapy, Klinikum rechts der Isar, School of Medicine, Technical University of Munich, Munich, Germany; ^2^Institute of Psychiatric and Psychosomatic Psychotherapy, Central Institute of Mental Health Mannheim, University Medical Center Mannheim, Mannheim, Germany

**Keywords:** anxiety, cancer, distress, fear of progression, fear of recurrence, psycho-oncology, worry

## Abstract

**Background:** Fear of progression (FoP), or fear of cancer recurrence (FCR), is characterized by worries or concerns about negative illness-related future events. Actually, to worry is a common cognitive process that, in its non-pathological form, belongs to daily life. However, worry can also become pathological appearing as a symptom of mental disorders. This study aimed at investigating the associations among daily worry, pathological worry, and FoP in patients with cancer.

**Methods:** This is a cross-sectional study that includes 328 hospitalized patients with cancer. Patients filled out the FoP Questionnaire (FoP-Q), the Worry Domains Questionnaire (WDQ) for the assessment of daily worry, and the Penn State Worry Questionnaire (PSWQ) for the assessment of pathological worry. Depressive, anxiety, and somatic symptoms were measured with modules of the Patient Health Questionnaire [Patient Health Questionnaire-Depressive Symptoms (PHQ-2), Generalized Anxiety Disorder-2 (GAD-2), and Patient Health Questionnaire-Somatic Symptoms (PHQ-15)]. Furthermore, a structured clinical interview was conducted for the assessment of anxiety disorders. The hierarchical multiple linear regression analysis was used to identify factors independently associated with FoP.

**Results:** Mean age of the participants was *M* = 58.5 years (SD = 12.8), and 64.6% were men. FoP and worry were significantly intercorrelated (*r* = 0.58–0.78). The level of FoP was most strongly associated with daily worry (*β* = 0.514, *p* < 0.001), followed by pathological worry (*β* = 0.221, *p* < 0.001). Further significant determinants were younger age and depressive and anxiety symptoms. Clinical variables were not independently associated with FoP. The final model explained 74% of the variance.

**Discussion:** Fear of progression is strongly associated with daily worry and pathological worry. These results bring up the question of whether FoP is an expression of a general tendency to worry. Whether a general tendency to worry, in fact, represents an independent vulnerability factor for experiencing FCR/FoP needs to be investigated in a longitudinal research design.

## Introduction

Many people experience recurrent thoughts about possible risks and threats. To think repetitively about such future uncertainties and dangers is quite common. In a study with community-dwelling elderly people, Golden et al. (2011) found that 78.7% of the respondents worried during the previous month. Furthermore, 37.1% stated that they worried excessively, and 20.0% experienced excessive uncontrollable worry. Such excessive and uncontrollable worry, but not non-severe worry, was associated with depression and reduced quality of life (Golden et al., 2011). Worry has been associated with several negative outcomes, including general anxiety disorder (GAD) as a manifestation of excessive and uncontrollable worry (Golden et al., 2011; Hirsch et al., 2013). Some researchers have also highlighted the positive functions of worry. Worry can act as a motivator and buffer (Sweeny and Dooley, 2017), it can reflect a constructive problem-solving process (Szabo and Lovibond, 2002), and it can facilitate goal pursuit and threat reduction (McNeill and Dunlop, 2016). Several measures for the assessment of worry were developed, focusing on the experience of daily worry (e.g., Worry Domains Questionnaire, WDQ; Tallis et al., 1992) as well as on the phenomenon of excessive pathological worry (e.g., Penn State Worry Questionnaire, PSWQ; Meyer et al., 1990).

Regarding specific worry topics, people tend to worry about interpersonal relationships, self, work, future events, finances, and mostly health (Tallis et al., 1992; Golden et al., 2011). For instance, it is quite common that healthy people worry about developing cancer (Jensen et al., 2010; Murphy et al., 2018).

For people who suffer from cancer, to worry about future uncertainties represents quite an adequate response as there are many real risks and threats in the disease course. Patients with cancer often worry about illness- and treatment-related aspects, e.g., the side effects of treatment or taking time away from the family (Pisu et al., 2017). However, most important is the worry about cancer progression or recurrence (Simard et al., 2013; Dinkel and Herschbach, 2018). Fear of cancer recurrence (FCR) has been defined as “fear, worry, or concern about cancer returning or progressing” (Lebel et al., 2016, p. 3266). Although nearly all patients and survivors with cancer experience FCR to some degree (Simard et al., 2013), an excessive FCR has been linked to several negative outcomes such as reduced quality of life and worse psychosocial wellbeing (Koch et al., 2013; Simard et al., 2013; Simonelli et al., 2017; Dinkel and Herschbach, 2018; Lebel et al., 2020). FCR and the very similar construct fear of progression (FoP) have been conceptualized as multidimensional (Herschbach et al., 2005; Simard and Savard, 2009). However, worry represents one central aspect in current conceptualizations of non-pathological and clinical FCR/FoP (Fardell et al., 2016; Mutsaers et al., 2016, 2020). In fact, some researchers solely focused on worry when assessing FCR (Vickberg, 2003; Custers et al., 2014).

Despite the prominent role of worry in the understanding and conceptualization of FCR/FoP, there have been few empirical attempts to link FCR/FoP to the literature on worry from the fields of clinical psychology and psychopathology. Some studies with patients with cancer used the PSWQ and investigated pathological worry as an outcome or mediator, showing that pathological worry can be reduced by a psycho-oncological intervention (Wells-Di Gregorio et al., 2019) and that worry mediates the effect of mindfulness on psychological distress (Labelle et al., 2015; Brown et al., 2020). Recent studies aiming at validating theoretical models of FCR assessed meta-cognitive beliefs about worry, but not worry itself (Lebel et al., 2018; Smith et al., 2018; Curran et al., 2020). Only one study investigated the association between FCR and pathological worry (PSWQ), showing that the two were moderately correlated (*r* = 0.49). Furthermore, clinical FCR was more frequent at higher levels of pathological worry (Hovdenak Jakobsen et al., 2018).

In a previous study, we investigated the comorbidity pattern between FoP and anxiety disorders in patients with cancer (Dinkel et al., 2014). We found that patients with pure clinical FoP (without comorbid anxiety disorder) did not differ from patients with pure anxiety disorder (without comorbid clinical FoP) regarding pathological worry. However, patients with cancer with pure clinical FoP showed even higher levels of daily worry than patients with pure anxiety disorder. Patients with comorbid clinical FoP and anxiety disorder indicated the highest levels of worry (Dinkel et al., 2014). These results suggest a prominent role of daily and pathological worry in FoP.

Thus, in this study, we investigated whether daily worry and pathological worry would be independently associated with FoP, controlling for well-known covariates as well as potential sociodemographic, clinical, and mental health covariates, i.e., variables with inconsistent or few positive findings with regard to FCR/FoP. Such results would be helpful for the empirical validation of current models of FoP/FCR (Fardell et al., 2016; Mutsaers et al., 2016, 2020).

## Methods

### Design and Procedure

This is a secondary analysis of the study by Dinkel et al. (2014). In brief, this was a cross-sectional investigation with patients with cancer undergoing inpatient treatment. Patients from the surgical or the hematological department of a large university hospital were sampled consecutively during 1 year (i.e., from March 2010 to March 2011). Inclusion criteria for study participation were confirmed diagnosis of gastrointestinal cancer or hematological malignancy, >18 years of age, and fluency with the German language. Exclusion criteria were severe psychiatric illness (except an anxiety disorder), severe physical, emotional, or cognitive impairment (rating of clinicians), and current treatment in the intensive care unit. Patients were approached by one of the authors (KK). Those patients who agreed to participate gave written informed consent. All participants underwent a structured clinical interview for the assessment of anxiety disorders and hypochondriasis and then filled out the self-reporting questionnaires. This study was approved by the local Ethics Committee (ethics vote: 2721/10).

### Measures

#### Sociodemographic and Clinical Characteristics

Sociodemographic characteristics were recorded using a documentation sheet. Medical records were assessed to extract the data on clinical characteristics. The functional status of patients was assessed using the Karnofsky Performance Status (KPS; Karnofsky and Burchenal, 1949) during the personal interview with the patient by one of the authors (KK). Furthermore, for the assessment of comorbidity, patients indicated whether they had been diagnosed with selected chronic conditions other than cancer.

#### Fear of Progression

The FoP Questionnaire (FoP-Q) by Herschbach et al. (2005) was used to measure FoP. This is a multidimensional, reliable, and valid measure (Thewes et al., 2012) that has been used in international research. The “coping with anxiety” subscale was not applied as this subscale does not contribute to the total score of the FoP-Q (see Herschbach et al., 2005). Thus, we presented 34 items that belong to one of the four subscales, namely, “affective reactions,” “partnership/family issues,” “occupation,” and “loss of autonomy.” Each item is rated on a 5-point scale ranging from 1 (never) to 5 (often). The total score is computed as the sum of the mean scores of subscales. Higher scores represent higher levels of FoP. Internal consistency in this study was α = 0.95. The 80th percentile of the FoP-summary score represented clinical FoP (Dinkel et al., 2014).

#### Worry

Two measures were applied for the assessment of worry.

The WDQ (Tallis et al., 1992; Stöber, 1995) was designed to measure non-pathological, daily worry. This is a content-oriented measure, asking participants to indicate on a 5-point scale from 0 (not at all) to 4 (extremely) how much they worry with regard to specific topics. The 25 items represent the five subscales, namely, “relationships,” “lack of confidence,” “aimless future,” “work incompetence,” and “financial.” A summary score ranging from 0 to 100 can be computed. Cronbach's alpha in this study was *α* = 0.95.

In contrast, the PSWQ (Meyer et al., 1990; Stöber, 1995) was developed to assess pathological worry, which is the main characteristic of GAD. The PSWQ represents a trait measure of the general tendency to worry excessively. It consists of 16 statements that do not relate to specific worry content but to the intensity and perceived uncontrollability of worry (e.g., “My worries overwhelm me”). Participants are instructed to indicate how typical the statements are for them. They responded on a 5-point scale ranging from 1 (not at all typical) to 5 (very typical). Five items are reverse scored. A total score, ranging from 16 to 80, is calculated by summing up all items. Cronbach's alpha in this study was α = 0.91.

For both measures, higher scores indicate higher levels of worrying.

#### Depression and Anxiety Symptoms

Symptoms of depression and anxiety were assessed using the ultra-short screening versions of the Patient Health Questionnaire (PHQ), i.e., PHQ-2 and GAD-2. Both modules comprise two items, which are rated on a 4-point scale from 0 (not at all) to 3 (nearly every day) (Löwe et al., 2010). Higher scores represent higher depression and anxiety. Internal consistency in this study was *α* = 0.82 (PHQ-2) and *α* = 0.80 (GAD-2).

#### Anxiety Disorder

In light of the aim of this study (see Dinkel et al., 2014), only anxiety disorders and hypochondriasis were assessed using the Structured Clinical Interview for DSM-IV Axis I (SCID-I) (Wittchen et al., 1997). The interviews were conducted by one coauthor (KK) who is a clinical psychologist trained in conducting SCID-I interviews.

#### Somatic Symptom Burden

The PHQ module, PHQ-15 (Kroenke et al., 2002; Kocalevent et al., 2013), was applied for the assessment of common somatic symptoms. Patients were asked to indicate the severity of 15 somatic symptoms during the previous 4 weeks. The symptoms were rated on a 3-point scale ranging from 0 (not bothered at all) to 2 (bothered a lot). Higher scores indicate a higher symptom load. Cronbach's alpha in this study was *α* = 0.80.

### Statistical Analysis

Descriptive statistics were computed for the study variables. The research question was investigated using the hierarchical multiple linear regression analysis. In order to control for known and potential covariates, sociodemographic characteristics were entered in the first step, clinical variables in the second step, and mental health variables in the third step. As our main study (Dinkel et al., 2014) showed that there is some overlap between anxiety disorder and FoP, we controlled for the presence of any anxiety disorder. However, as the presence of clinician-defined anxiety disorder and patient-reported anxiety symptoms do not correspond perfectly, we decided to control for both self-reported mental health symptoms and clinician-defined anxiety disorder. In the fourth step, the full model is presented. The full model includes the following variables: age, gender, current partnership, educational level, cancer site, disease status, duration of disease, functional status, comorbidity, depressive symptoms, anxiety symptom, somatic symptoms, anxiety disorder, daily worry, and pathological worry. Within each step, variables were entered simultaneously. We presented the adjusted *R*^2^ as the measure of variance explained as well as Δ*R*^2^ indicating the change in *R*^2^ between each step of the hierarchical regression. The following variables were dichotomized for the regression analysis: educational level (lower/higher), cancer site (gastrointestinal/hematological), comorbidity (none/present), and disease status (first occurrence/all others). For additional analyses, Pearson's correlations were used to assess intercorrelations between worry and FoP, and differences in worry mean scores between groups of clinical versus non-clinical FoP were investigated using the independent sample *t*-test. Effect sizes (Cohen's *d*) are reported for these group differences. Alpha level was set as *p* < 0.05. All analyses were conducted using SPSS/PC software package version 24 (SPSS, Chicago, IL, USA).

## Results

### Study Sample

Of 529 patients who were approached for participation, 49 patients met the exclusion criteria. Thus, 480 patients were available for study participation. A total of 343 patients (71.5%) agreed to take part. Patients who declined participation did not differ from the study participants with regard to sex or cancer site, but patients who declined were older (*M* = 63.1, SD = 11.6) than those patients who agreed (*p* < 0.001). Of those who agreed, 15 patients did not provide the data on FoP, leaving 328 patients available for the analysis. In light of the low number of patients who were excluded from the analysis, we refrained from conducting a drop-out analysis.

The mean age of the patients was *M* = 58.5 years (SD = 12.8; minimum–maximum: 20–87). The majority of them (64.6%) were men. A total of 60.7% of the patients suffered from gastrointestinal cancer (mainly colorectal cancer, *n* = 81) and 39.3% suffered from hematological malignancy (mainly lymphoma, *n* = 43). For most of the patients, this was the first occurrence of the disease (69.0%), and the mean time since the first cancer diagnosis was 27.1 months (SD = 56.8; minimum–maximum: 0–546 months). Further sociodemographic and clinical characteristics are presented in Table 1.

**Table 1:** Sociodemographic and clinical characteristics (*N* = 328).

	**M**	**SD**	**n**	**%**
Age, years (*n* = 328)	58.5	12.8		
Sex (*n* = 328)				
Men			212	64.6
Women			116	35.4
Marital status (*n* = 328)				
Single			54	16.5
Married			222	67.7
Divorced			35	10.7
Widowed			17	5.2
Current partnership (*n* = 326)				
Yes, living together			246	75.5
Yes, living apart			28	8.6
None			52	16.0
Children (*n* = 326)				
Yes			237	72.7
None			89	27.3
Educational status (*n* = 324)				
Elementary school			121	37.3
Secondary school/junior high			69	21.3
High school			118	36.4
Other			16	4.9
Employment status (*n* = 327)				
Full-time			94	28.7
Less than full-time			27	8.2
Unemployed			16	4.9
Homemaker			13	4.0
Retired/Disability pension			141	43.1
Other			36	11.0
Net household income (*n* = 251)				
< 1000 Euro			32	12.8
< 2000 Euro			68	27.1
< 3500 Euro			94	37.4
> 3500 Euro			57	22.7
Subjective economic situation (*n* = 324)				
Very good			34	10.5
Good			143	44.1
Satisfactory			123	38.0
Not so good			15	4.6
Poor			9	2.8
Cancer site (*n* = 328)				
Gastrointestinal			199	60.7
Hematological			129	39.3
Disease status (*n* = 326)				
First occurrence			225	69.0
Second/third primary cancer			33	10.1
Recurrence			39	12.0
Complete/partial remission			23	7.0
Unknown			6	1.8
Treatment intent (*n* = 282)				
Curative			214	75.9
Palliative			68	24.1
Current treatments (*n* = 327)				
Surgery			138	42.2
Chemotherapy			110	33.6
Immunotherapy			1	0.3
Stem cell transplantation			20	6.1
In planning			19	5.8
Aftercare			14	4.3
Other			25	7.6
Duration of the disease^a^, months (*n* = 315)	27.1	56.8		
Functional status (KPS^b^) (*n* = 328)				
≤ 60			22	6.7
≤ 70			48	14.6
≤ 80			52	15.9
≤ 90			90	27.4
> 90			116	35.3
Comorbid chronic condition^c^ (*n* = 315)				
Yes			93	29.5
None			222	70.5
Anxiety disorder (*n* = 328)				
Yes			60	18.3
None			268	81.7

*Data may not be summed to the full sample size due to missing data*.

a *Duration since first cancer diagnosis*.

b *KPS, Karnofsky Performance Status*.

c *This category includes the following diagnoses: asthma, bipolar disorder (former episode), chronic kidney disease, chronic obstructive pulmonary disease, coronary heart disease, Crohn's disease, depression (current and former episode), diabetes mellitus, epilepsy, fibromyalgia, HIV, hyperthyroidism, liver cirrhosis, multiple sclerosis, Parkinson's disease, rheumatoid arthritis, and ulcerative colitis*.

### Bivariate Associations

Descriptives of the continuous variables are given in Table 2. Regarding intercorrelations, the results revealed that FoP correlated *r* = 0.78 with daily worry (WDQ) and *r* = 0.64 with pathological worry (PSWQ). The two worry measures correlated *r* = 0.58 (all correlations *p* < 0.001).

**Table 2:** Mean (M) and standard deviation (SD) of study variables.

**Variable**	**M**	**SD**
Fear of progression (FoP-Q) (*n* = 328)	7.1	2.6
Daily worry (WDQ) (*n* = 318)	13.1	14.8
Pathological worry (PSWQ) (*n* = 312)	37.4	11.3
Depressive symptoms (PHQ-2) (*n* = 327)	1.3	1.6
Anxiety symptoms (GAD-2) (*n* = 327)	1.6	1.6
Somatic symptoms (PHQ-15) (*n* = 327)	8.2	5.5

*FoP-Q, Fear of Progression Questionnaire; WDQ, Worry Domains Questionnaire; PSWQ, Penn State Worry Questionnaire; PHQ-2, Patient Health Questionnaire-Depressive Symptoms; GAD-2, Generalized Anxiety Disorder-2; PHQ-15, Patient Health Questionnaire-Somatic Symptoms*.

**Figure 1:**
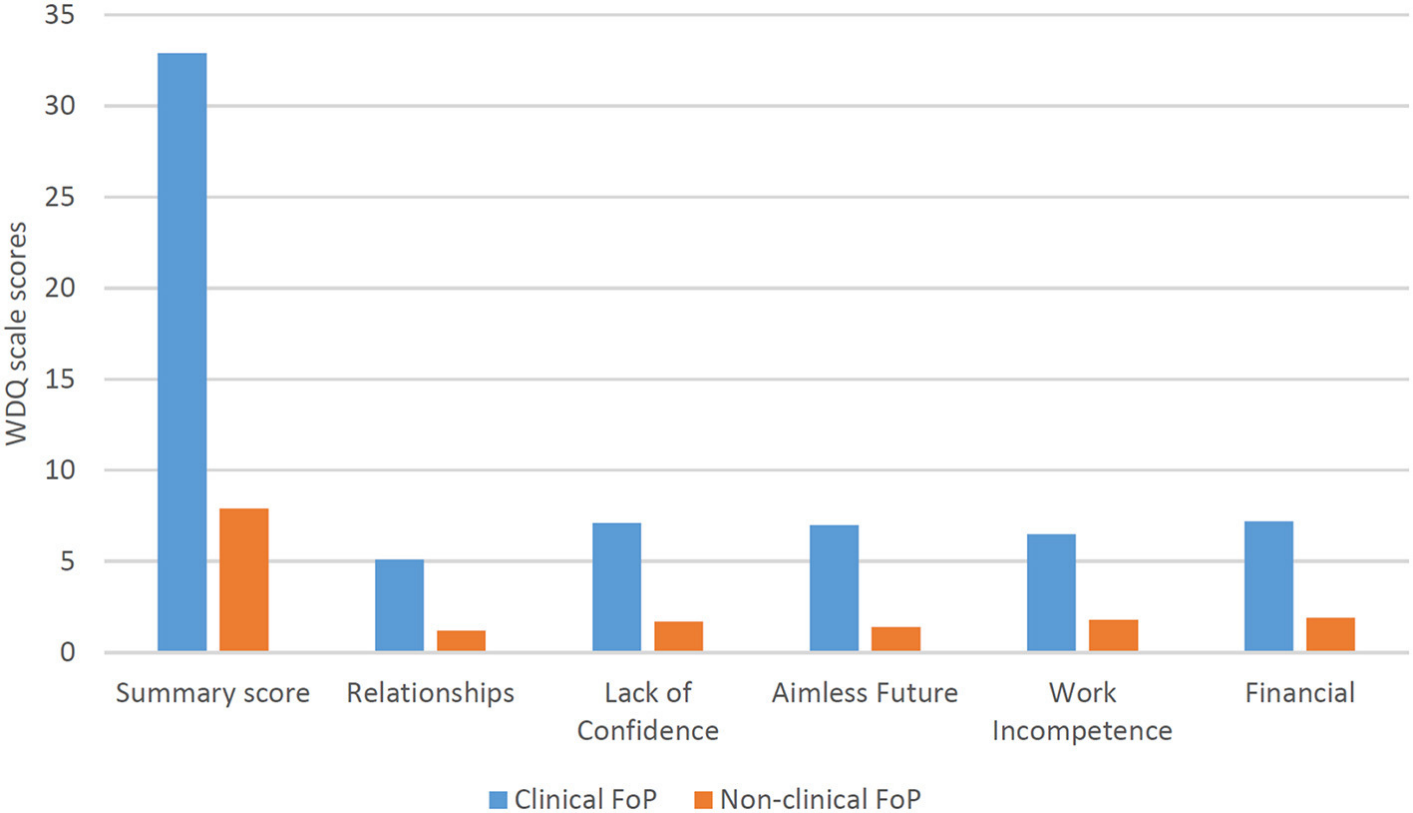
Differences in the scales of the Worry Domains Questionnaire between patients with cancer with clinical versus non-clinical fear of progression (FoP-Q; cut off: 80th percentile).

**Figure 2:**
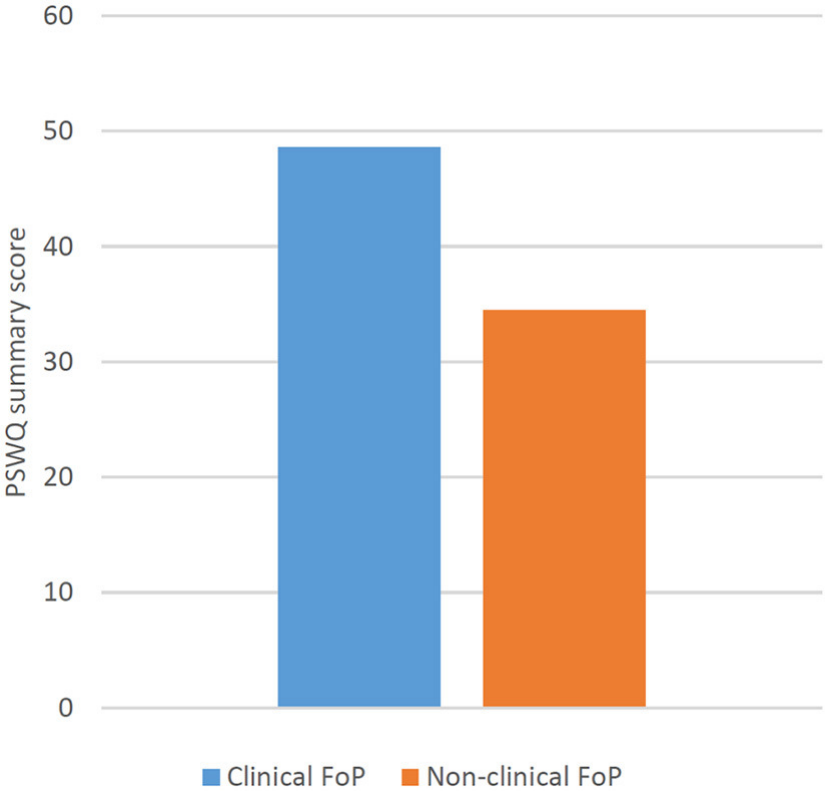
Differences in the summary scale score of the Penn State Worry Questionnaire between patients with cancer with clinical versus non-clinical FoP (FoP-Q; cut off: 80th percentile).

Patients with clinical FoP (*n* = 66) showed significantly higher (*p* < .001) daily worry (Figure 1) as well as pathological worry (Figure 2) [see also Supplementary Material 1].

### Multiple Linear Regression

We conducted a hierarchical multiple linear regression analysis to identify the determinants of FoP. The sociodemographic data were entered in the first step. The results showed that younger age and lower educational level were significantly associated with FoP. While age remained a significant determinant until the last step, the educational level lost significance in the third step when variables representing psychological distress were entered. Regarding clinical variables, which were entered in the second step, the results showed that only lower functional status was significantly associated with FoP. However, this association became non-significant in the third step. In the third step, depressive, anxiety, and somatic symptoms showed a significant association with FoP.

Finally, in the last step, daily worry and pathological worry were entered into the regression. Controlling for sociodemographic, clinical, and mental health variables, both daily worry and pathological worry were independently associated with FoP. In fact, the two worry variables showed the highest beta weights of all variables, with daily worry being most strongly associated with FoP (*β* = 0.514, *p* < 0.001). The final regression model revealed that younger age, current partnership, higher anxiety, depressive, and somatic symptoms, absence of an anxiety disorder, and higher worry were significant determinants of FoP (Table 3). The final regression model explained 74% of the variance.

**Table 3:** Results of the hierarchical multiple regression analysis predicting fear of progression (FoP-Q) (*n* = 281).

**Step**	**Variable**	**B**	**SE B**	**Beta**	***p***	**Adj. *R^**2**^***	**Δ** ***R*** ^**2**^	**p Δ *R*^**2**^**
1						0.093		
	Age	−0.053	0.011	−0.268	< 0.001			
	Gender	0.429	0.305	0.081	0.161			
	Current partnership	0.700	0.403	0.100	0.084			
	Educational level	−0.723	0.300	−0.137	0.017			
2						0.119	0.026	0.026
	Age	−0.063	0.013	−0.319	<0.001			
	Gender	0.403	0.306	0.076	0.189			
	Current partnership	0.788	0.407	0.112	0.054			
	Educational level	−0.758	0.298	−0.144	0.012			
	Cancer site	0.147	0.310	0.028	0.636			
	Disease status	−0.038	0.352	−0.007	0.915			
	Duration of the disease	0.003	0.003	0.058	0.360			
	Functional status	−0.040	0.012	−0.198	0.001			
	Comorbidity	0.004	0.348	0.001	0.990			
3						0.467	0.348	<0.001
	Age	−0.045	0.010	−0.228	<0.001			
	Gender	−0.119	0.242	−0.023	0.621			
	Current partnership	0.863	0.322	0.123	0.008			
	Educational level	−0.249	0.236	−0.047	0.293			
	Cancer site	0.106	0.242	0.021	0.661			
	Disease status	0.082	0.276	0.015	0.767			
	Duration of the disease	0.004	0.002	0.082	0.101			
	Functional status	0.005	0.010	0.024	0.632			
	Comorbidity	−0.222	0.273	−0.038	0.417			
	Depressive symptoms	0.388	0.101	0.244	<0.001			
	Anxiety symptoms	0.467	0.103	0.294	<0.001			
	Somatic symptoms	0.099	0.026	0.215	<0.001			
	Anxiety disorder	0.176	0.305	0.027	0.564			
4						0.739	0.272	<0.001
	Age	−0.022	0.007	−0.109	0.003			
	Gender	0.029	0.170	0.005	0.865			
	Current partnership	0.782	0.226	0.112	0.001			
	Educational level	−0.184	0.166	−0.035	0.268			
	Cancer site	−0.093	0.170	−0.018	0.584			
	Disease status	0.227	0.193	0.041	0.240			
	Duration of the disease	0.003	0.002	0.062	0.073			
	Functional status	−0.004	0.007	−0.020	0.562			
	Comorbidity	−0.193	0.191	−0.033	0.313			
	Depressive symptoms	0.203	0.072	0.127	0.005			
	Anxiety symptoms	0.208	0.076	0.131	0.007			
	Somatic symptoms	0.203	0.072	0.061	0.129			
	Anxiety disorder	−0.515	0.218	−0.080	0.019			
	Daily worry	0.088	0.007	0.514	<0.001			
	Pathological worry	0.049	0.010	0.221	<0.001			

*Coding of dichotomous variables: gender: men = 0; current partnership: none = 0; educational level: lower = 0; cancer site: gastrointestinal = 0; comorbidity: none = 0; disease status: first occurrence = 0; anxiety disorder: none = 0*.

## Discussion

Patients who suffer from a chronic disease often experience fears that relate to the illness and its biopsychosocial consequences (Berg et al., 2011; Lebel et al., 2020). FoP and FCR represent adequate psychological responses to real threats and risks of the cancer experience that, nonetheless, can become dysfunctional (Simonelli et al., 2017; Dinkel and Herschbach, 2018; Lebel et al., 2020). The main feature of current conceptualizations and definitions of FCR/FoP is worry (Fardell et al., 2016; Mutsaers et al., 2016, 2020). Worry is common in daily life, but it can also occur at degrees that can be characterized as pathological (Golden et al., 2011). Interestingly, these different lines of research—psycho-oncology on the one hand, and clinical psychology and psychopathology on the other hand—have not met with regard to worry and FCR/FoP. In this study, we aimed at connecting these different lines of research, investigating whether daily worry and pathological worry would be independently associated with FoP.

As a main result, our analysis revealed that daily worry and pathological worry were the most relevant determinants of FoP, controlling for sociodemographic variables, clinical characteristics, and symptom burden, i.e., patients with cancer who indicated a high level of worry in a measure designed for the assessment of non-pathological worry and who indicated to experience a high amount of excessive and uncontrollable worry reported higher levels of FoP. These results suggest that patients who are characterized by a general tendency to worry are more prone to experience FoP when diagnosed with cancer. Clearly, the cross-sectional nature of this study precludes strong inferences regarding causal or longitudinal associations. Thus, whether a general tendency to worry, in fact, represents an independent vulnerability factor for experiencing FCR/FoP needs to be investigated in a longitudinal research design. Nonetheless, these results fit very well with the current conceptualizations of FCR, which include meta-cognitive beliefs about worry (Fardell et al., 2016; Lebel et al., 2018) and a cognitive-attentional syndrome, characterized by worry, rumination, and attentional bias to threat-related information (Fardell et al., 2016).

In an attempt to derive not only at a conceptualization of FCR but also on a theoretical model of anxiety in the context of cancer, Curran et al. (2017) reviewed the literature and developed a model quite similar to that by Fardell et al. (2016), which relates to FCR. Curran et al. (2020) tested some of the assumptions of this general model of anxiety in patients with cancer with regard to FoP as an outcome. In this cross-sectional study with 211 patients with cancer, the authors investigated the association of rumination, assessed by a measure of transdiagnostic repetitive thinking, with FoP. These two variables correlated *r* = 0.60, but rumination—or repetitive thinking—did not emerge as an independent determinant of FoP after death anxiety, intrusions, and threat appraisal had been entered into the regression. Worry and rumination—or repetitive thinking—share relevant features, thus both this study as well as the study by Curran et al. (2020) underscore the strong association between repetitive cognitive processes and the experience of FoP. In this study, worry emerged as the most relevant determinant of FoP. However, in contrast to the study by Curran et al. (2020), we did not investigate the role of death anxiety, intrusions, or threat appraisal. Thus, it remains to be shown whether worry will be an independent vulnerability factor for FCR/FoP.

According to several reviews of FCR/FoP (Crist and Grunfeld, 2013; Simard et al., 2013; Dinkel and Herschbach, 2018; Lim and Humphris, 2020), lower age and higher somatic symptom burden represent the most consistent predictors of higher levels of FCR/FoP. While the effect of age was replicated in this study, the association between somatic symptoms and FoP disappeared after the inclusion of worry. In accordance with available evidence (Simard et al., 2013), depressive and anxiety symptoms were also significantly associated with FoP in this study. Likewise, in accordance with other studies (Simard et al., 2013; Smith et al., 2018), clinical characteristics were not independently associated with FoP. Interestingly, we found that the absence of an anxiety disorder represented a determinant of FoP. However, this effect was quite weak (*β* = −0.08), and the significance of this finding remains unclear.

Finally, our results also support some current psycho-oncological interventions addressing FCR/FoP. A recent systematic review and meta-analysis showed that psycho-oncological interventions are effective in reducing FCR/FoP (Tauber et al., 2019). This meta-analysis also revealed that contemporary cognitive-behavioral therapies, i.e., approaches that focus on cognitive processes—like worry or rumination—were more effective, at least in the short run, than traditional cognitive-behavioral approaches, defined as those interventions that focus primarily on the content of cognition. This view is supported by the independent association between pathological worry and FoP, as we assessed pathological worry with a content-free measure that focused on the intensity and perceived uncontrollability of worry. However, our results also support approaches focusing on the content of cognition, like our own therapeutic approach, which applies exposure-based techniques. Similar to the cognitive-behavioral approach for GAD, which focuses on the exposure of worry themes, patients with cancer are asked to vividly recount their worries and to work through a worst-case scenario (Dinkel and Herschbach, 2018). This approach has proven feasible and effective in reducing FoP of patients with cancer (Herschbach et al., 2010; Dinkel et al., 2012; Rudolph et al., 2018) and is supported by the independent association between our measures of daily worry, which focuses on the content of worrying thoughts.

This study has some strengths, such as the detailed assessment of worries in patients with cancer, the reasonable sample size, and the inclusion of a set of covariates. But, clearly, it also has some limitations. First, the cross-sectional design precludes inferences about longitudinal associations between worry and FoP. Then, there is a sampling bias as patients who declined participation were older than those who agreed to take part. Furthermore, we did not assess other psychological variables that have proven relevant as possible control variables, especially those that are regarded as important in current theoretical conceptualizations of FCR/FoP, e.g., meta-cognitive beliefs. Moreover, we did not assess the whole spectrum of mental disorders but restricted our assessment on the anxiety disorders. Finally, there might be a bias due to shared method variance. We applied three self-reporting measures focusing on different aspects of worry that were moderately to highly intercorrelated. Thus, the results of this study should be replicated using different assessment approaches.

## Conclusion

This study has shown that worry represents an independent determinant of FoP. As such, the results support current theoretical conceptualizations of non-clinical and clinical FCR/FoP (Fardell et al., 2016). However, these results also bring up the question of whether FoP is an expression of a general tendency to worry. Thus, associations between repetitive cognitive processes and FCR/FoP should be investigated further in future studies.

## Data Availability Statement

The datasets presented in this article are not readily available because participants did not provide consent for public availability of the data. Requests to access the datasets should be directed to Andreas Dinkel, a.dinkel@tum.de.

## Ethics Statement

The studies involving human participants were reviewed and approved by Ethics committee of the Faculty of Medicine, Technical University of Munich. The patients/participants provided their written informed consent to participate in this study.

## Author Contributions

AD designed the study. KK was responsible for data acquisition. KK and BM-M prepared and analyzed the data. AD wrote the first draft. All authors contributed significantly to the interpretation of the data and the final version of the manuscript and gave final approval of the version to be published.

## Conflict of Interest

The authors declare that the research was conducted in the absence of any commercial or financial relationships that could be construed as a potential conflict of interest.

## Publisher's Note

All claims expressed in this article are solely those of the authors and do not necessarily represent those of their affiliated organizations, or those of the publisher, the editors and the reviewers. Any product that may be evaluated in this article, or claim that may be made by its manufacturer, is not guaranteed or endorsed by the publisher.
