# Supplementary material for: Association Between Daily Worry, Pathological Worry, and Fear of Progression in Patients With Cancer
Source: Front Psychol. 2021 Aug 11;12:648623. doi: 10.3389/fpsyg.2021.648623 (PMC8384960; doi:10.3389/fpsyg.2021.648623)
Supplement: Supplementary file 1 [file Table_1.docx]

**Supplementary Table**

**Table S1**: Comparison of means (t-test), patients with clinical vs. non-clinical fear of progression (FoP-Q)

|  | **Clinical FoP** | | **Non-clinical FoP** | | **p** | **d** |
| --- | --- | --- | --- | --- | --- | --- |
|  | **M** | **SD** | **M** | **SD** |  |  |
| WDQ-Summary scale | 32.9 | 16.9 | 7.9 | 8.5 | <.001 | 2.3 |
| WDQ-Relationships | 5.1 | 3.7 | 1.2 | 1.7 | <.001 | 1.7 |
| WDQ-Lack of Confidence | 7.1 | 3.9 | 1.7 | 2.4 | <.001 | 1.9 |
| WDQ-Aimless Future | 7.0 | 4.0 | 1.4 | 1.9 | <.001 | 2.3 |
| WDQ-Work Incompetence | 6.5 | 4.3 | 1.8 | 2.2 | <.001 | 1.7 |
| WDQ-Financial | 7.2 | 5.5 | 1.9 | 2.8 | <.001 | 1.5 |
| PSWQ-Summary scale | 48.6 | 11.7 | 34.5 | 9.2 | <.001 | 1.4 |

Note: FoP-Q – Fear of Progression Questionnaire; WDQ – Worry Domains Questionnaire; PSWQ – Penn State Worry Questionnaire; sample size: n = 318 (WDQ) and n = 312 (PSWQ); d – Cohen's d (effect size)
